# SimUniversity at a distance: a descriptive account of a team-based remote simulation competition for health professions students

**DOI:** 10.1186/s41077-021-00199-5

**Published:** 2022-02-08

**Authors:** Stella Major, Ralf Krage, Marc Lazarovici

**Affiliations:** 1 Present Address: Division of Medical Education, Weill Cornell Medicine- Qatar,; 2 Department of Anesthesiology and Intensive Medicine, KJF Klinik St. Elisabeth, Neuburg, Germany; 3 University Hospital Munich, Institute for Emergency Medicine and Management in Medicine – INM, Munich, Germany

**Keywords:** Remote simulation, Competition, Non-technical skills, Interprofessional, Virtual debriefing

## Abstract

**Background:**

SimUniversity competition is an innovative Society in Europe for Simulation Applied to Medicine (SESAM) initiative which has existed since 2014, with the aim of creating opportunities for undergraduate healthcare students to take part in a formative educational experience on an international platform. The main educational focus is on promoting non-technical skills such as leadership, situation awareness, decision making, communication, and assertiveness, but also clinical reasoning within a team. In preparation for the 2021 virtual conference, the team designed a new methodology to meet the same mission, and yet be offered remotely.

**Main text:**

In this article, we describe the way in which we transformed the SimUniversity competition activity from face to face to a remote simulation. We relied on Zoom as the main communication technology to enable the distance component and followed the key elements of pre-briefing, simulation, and debriefing with the students being onsite together in one location and the faculty and simulator technologists in distant locations. Thirty-eight medical and nursing students formed 8 teams from 7 different countries. Two participating teams were based in Germany and one in Italy, Belgium, the Netherlands, Romania, Portugal, and Syria. Each team consisted of between 4 and 5 members and was self-selected to consist of either medical students alone or medical and nursing students together. The SimUniversity faculty team was composed of 5 physician educators, one nurse educator, one paramedic simulation technologist, and one industry simulation technologist. The faculty members facilitated each simulation synchronously in Zoom, while being based in different geographical locations within Europe (Germany, Switzerland, and the Netherlands) and the Middle East (Qatar and Lebanon).

**Conclusion:**

We conclude that assuming there is access to adequate internet connectivity and minimal technical setup, conducting a remote simulation with virtual debriefing is achievable in supporting team-based learning, particularly when learners and/or faculty members are in distant locations. While the authors do not recommend this method to be superior to a face-to-face experience, we propose this model to be an alternative method to consider when educators are faced with imposed restrictions such as what we faced during the COVID-19 pandemic. We discuss lessons learned and highlight other potential benefits that this method may provide, to consider even when the restrictions are lifted.

**Supplementary Information:**

The online version contains supplementary material available at 10.1186/s41077-021-00199-5.

## Background

Since 2014, SimUniversity has been an innovative initiative of the Society in Europe for Simulation Applied to Medicine (SESAM). It aimed at creating opportunities for undergraduate healthcare student teams (medical, nursing, and paramedic students) to take part in a formative educational experience on an international platform [1]. SESAM, a not-for-profit society founded in 1994, has its main goal to encourage and support the use of simulation in healthcare for the purpose of training and research.

Dong et al. conducted a qualitative descriptive study of emergency medicine residents who were participating in SimWars, an on-stage simulation competition assessing teamwork, communication, and patient care. They expressed the value of competition as a motivator for participants to do their best [2]. In light of this same spirit, over the last 7 years, SimUniversity competitions have offered teams of undergraduate students a chance to compete in simulation scenarios, and engage in debrief conversations under the supervision of experienced clinical and simulation experts.

All sessions are specifically designed to provide a safe, educational, and enjoyable learning environment [3]. The overall spirit of the SESAM SimUniversity is mainly fun and educational with a little flavor of competition. In SimUniversity, the scenarios mirror basic emergency situations (e.g., resuscitation, anaphylaxis, myocardial infarction) reflecting the students’ level of knowledge/skills. Because those skill sets could potentially differ from team to team (different national curricula, different cultures) after every scenario, students are debriefed by expert facilitators, with the focus being on promoting non-technical skills such as leadership, situational awareness, decision making, communication, and assertiveness, in addition to clinical reasoning within a team.

**Fig. 1:**
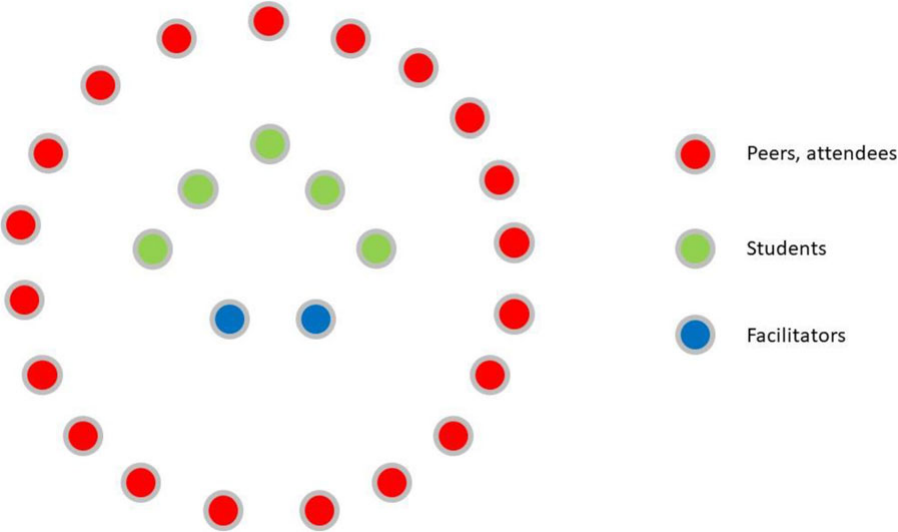
Three-tiered fishbowl representing the diverse learning opportunities of SimUniversity

A unique aspect of the SimUniversity is that the finalists’ simulations typically occur during the annual SESAM simulation conference. Thus, the competition offers a wide array of learning opportunities to diverse learners. Figure 1 illustrates how in each of the final simulation and live debriefings students and conference delegates (peer students, attendees) can benefit from and learn more about how to conduct simulations and debriefings, as they watch and listen to each of the simulations and live debriefings.

Due to the COVID-19 pandemic, the SESAM 2021 conference was delivered virtually, and in this regard, the SimUniversity planning committee also decided to develop an alternative methodology which would conform to the requirements of a virtual conference while keeping all elements of the in-person event intact.

According to the Healthcare Simulation Dictionary V2.0 Addendum, a remote simulation is “a simulation performed with either the facilitator, learners, or both in an offsite location separate from the other members to complete educational or assessment activities [4]. Facilitation and assessment can be performed either synchronously or asynchronously using video or web conferencing tools [5].”

To our knowledge, there is no report published in the literature which describes how to conduct a competition using remote simulation within a virtual conference. This paper describes how the authors developed and conducted team-based remote simulation including virtual debriefings as defined by Cheng et al. [3] to undergraduate healthcare students during the SESAM 2021 virtual conference. We describe the elements that educators may choose to consider, ranging from technical setup to actual simulations, and conclude with lessons learned, drawing upon participant feedback and the SimUniversity faculty reflections.

## Methods

### Overview of a SimUniversity session

While there are numerous different technological setups to consider, we prioritized inclusivity over fidelity meaning that for a team to participate in the competition, they were required to have access to any adult-sized manikin, so that they could perform technical hands-on tasks such as chest compression and airway management during the scenario. The manikin was however switched off, and the patient’s vital signs were controlled remotely by a technologist and screen shared to the students in Zoom. Referring to the key elements of simulation as described by Diaz et al. Table 1 describes the SimUniversity telesimulation competition [5].

**Table 1:** Overview of key elements of the SimUniversity telesimulation competition

Element	Descriptor
Student orientation	Students were encouraged to choose a location well known to them for the simulation. Manikin, medical equipment, and the environment were thus familiar to the participants.
Simulator type	In order to be as inclusive as possible, we did not require any specific type nor manufacturer for the manikin to be used.
Simulation environment	The simulations were conducted online in a synchronous manner. Students were all gathered together at their location of choice, usually a simulation center or some other learning location. The facilitators and the technician were all in separate locations. Everyone was connected through Zoom, the online meeting platform.
Simulation scenario	All 8 teams were offered scenario 1. Scenario 1: An adult patient was brought to the emergency room with a cardiac arrest and each team was required to perform advanced life support (ALS). Four finalist teams were offered scenario 2. Scenario 2: A young adult patient was brought to the emergency room with altered mental status (AMS). Each team had to evaluate the different differential diagnoses using a structured ABCDE approach.
Instructional design or exposure	In order to most closely resemble the classical face-to-face simulation competition, we used the traditional sequence of briefing, team-based simulated of the case, and virtual debriefing. *Duration:* The case was planned to run over approximately 10 min and the debriefing over 15–20 min.
Debriefing	Debriefing was focused on non-technical skills, clinical reasoning, and teamwork. As the simulator (manikin) was switched off, all clinical examination findings were communicated to the students, via an overhead intercom (colloquially also referred to as “Voice of God”). Given that all clinical actions had a level of abstraction built into the simulation, discussion of the clinical findings was not the main focus of the debriefing. We chose PEARLS [6] as the debriefing structure, combined with the advocacy/inquiry technique [7] during the analysis phase.

**Fig. 2:**
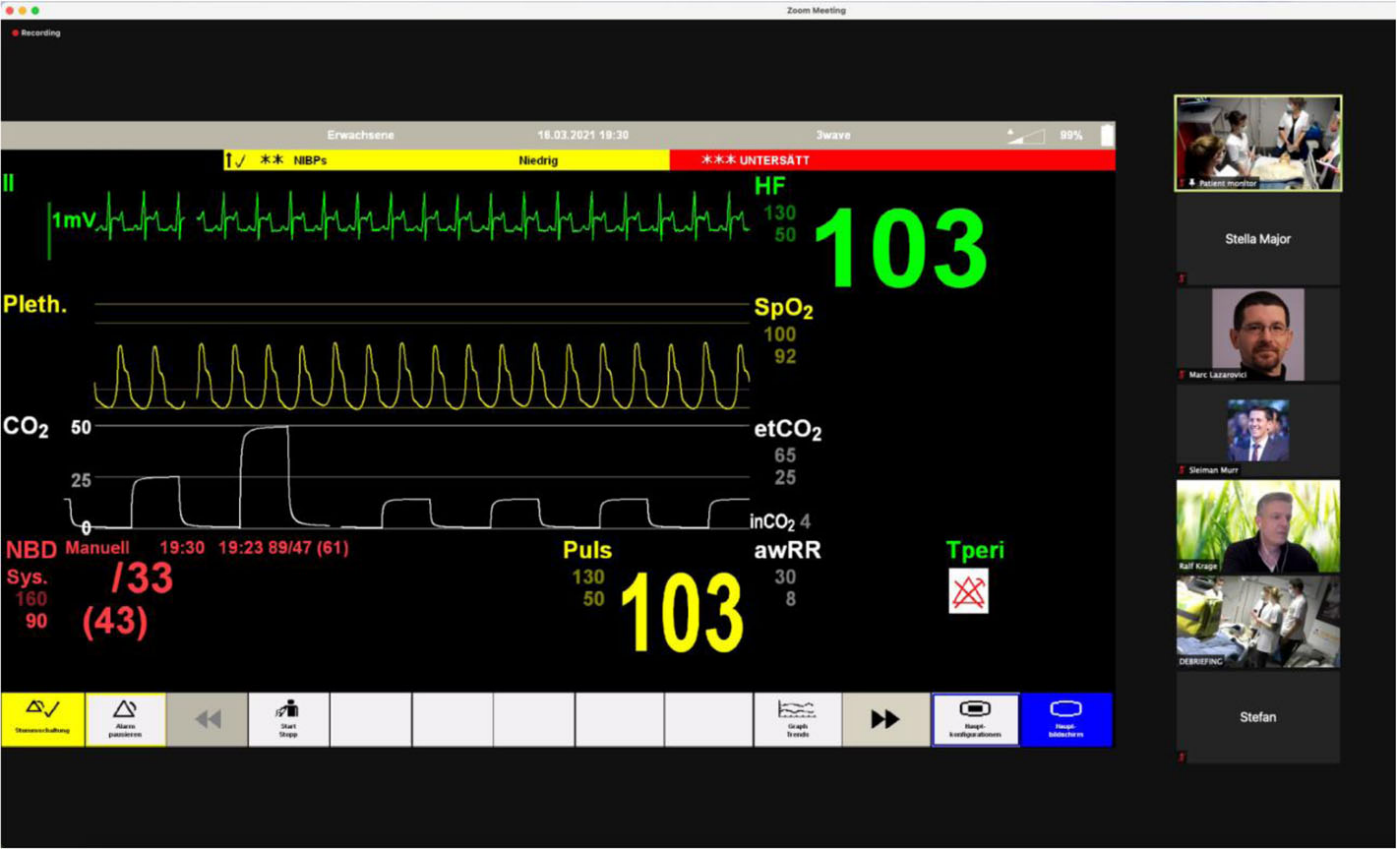
Patient monitor as was visible to the students in the simulation room

In line with the outline proposed by Duff et al. in our remote simulation setup, the communication technology used to enable the distance element was Zoom, and the elements of simulation were prebriefing, simulation, and debriefing [8]. We selected LLEAP (Laerdal Medical) as the software to generate the patient’s vital signs and set it on “virtual manikin mode” (Table 2). This was operated by a technician outside the center and was projected to the participants in their center via Zoom (Fig. 2).

**Table 2:** Configuration of distance elements

	In-person in the center (all in one space)	Outside the center (synchronous audiovisual)
**Active learners (students)**	**X**	
**Facilitators/debriefers**		**X (separated geographically)**
**Manikin**	**X**	
**Operator with LLEAP**		**X**

To be as inclusive as possible, we opted to have no connection between the LLEAP software and the physical manikin which the students had to use in their location to participate in the simulation competition. Had we considered connecting the LLEAP software with a remote manikin, this would have meant that all student teams would have been compelled to access a Laerdal product, in order to be eligible to participate.

### Participants

#### Student teams

The International Federation of Medical Students Associations (IFMSA), the European Medical Students Association (EMSA), and the SESAM SimUniversity community networks advertised this event and encouraged health profession students to apply and take part in this annual simulation competition. Thirty-four medical and 4 nursing students from 7 different countries participated in the SimUniversity 2021 event. A total of 8 teams competed, with 2 coming from Germany and one team from Italy, Belgium, the Netherlands, Romania, Portugal, and Syria. Each team size ranged between 4 and 5 members, and each team self-selected to be either members of one profession (medicine only) or interprofessional (medicine and nursing).

#### SimUniversity faculty

The SimUniversity faculty team was composed of 5 physician educators, one nurse educator, one paramedic simulation technologist, and one industry simulation technologist. Throughout the entire process, the team members participated from different geographical locations across Europe (Germany, Switzerland, and the Netherlands ) and the Middle East (Qatar and Lebanon).

### SimCompetition

After the identification of the participating student teams, a preliminary information sheet was sent to all the teams to inform them of the equipment and technology requirements (Appendix). A brief technical check session was scheduled for each team in Zoom and required them to be at the same location from which they would be participating in the competition. In this session, students were assisted in setting up the audio, technology, manikin, and cameras in a way that would permit the remote simulation to be achieved effectively and to ensure that the simulation could be recorded and shown during the SimUniversity competition sessions in the virtual conference. Twelve remote simulations were conducted over a period of 1 month, from March to April 2021.

### Session participants and roles and responsibilities

The facilitators and back office operators were pre-assigned specific roles. The 4 roles were as follows:

1. Designated session moderator
2. Overhead intercom and “voice of patient” responder
3. Laerdal Learning Application (LLEAP) operator
4. Session recorder in Zoom

Two simulation facilitators (lead and co-lead) led the session and were always audible and visible to the students in Zoom. The “back office operators” were the technical operators, and other SimUniversity faculty team members were the observers and supported the session where necessary. They met with the students at the introduction phase only and then were muted and invisible to the students (camera off).

### Overview of a SimUniversity session

Every session was divided into three different sections:

1. Preliminary pre-brief, setting of ground rules (around 15 min). This phase served to establish all the rules pertaining to a remote simulation session, including psychological and physical safety as well as technical aspects. During this phase, learners’ expectations were also managed, as has been recommended by other authors as well [8, 9].
  - Introductions: Everyone on the call briefly introduced themselves. The second intention of this part was for the debriefers to be able to differentiate the participants by optical clues and be able to address them in person, during the debriefing.
  - Goal exposition: The overall goals of the session were reiterated clearly as follows: that it is an interactive learning activity, which focuses on technical/non-technical skills, and not only on the scenario but more importantly on the debriefing.
  - Consent on video recording: Due to the nature of the session, this was done by running a visual/oral consent with thumbs up by the participants.
  - Simulation ground rules: To keep the manikin inoperative, communicate only via Zoom and respect all safety measures when using any real devices.

#### Scenario (10 min)

**Fig. 3:**
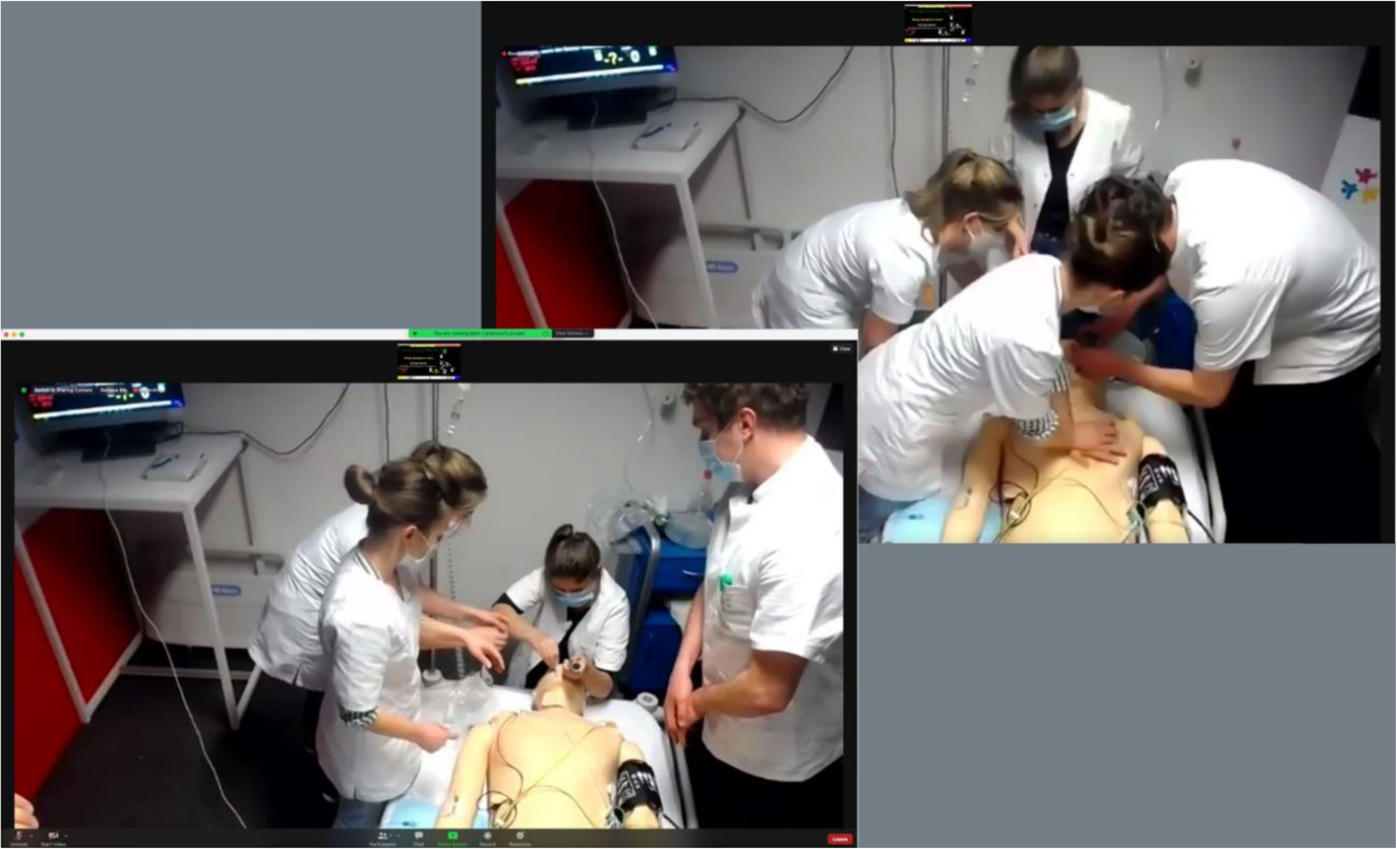
Team activity, as was visible to the facilitators (several different camera perspectives offered multiple views)

The team was encouraged to perform any activity that would result in findings (e.g., auscultation) and was urged to ask the faculty about any findings only after performing the activity. Any information which was required was provided by the faculty facilitators through the overhead intercom (Fig. 3).

**Fig. 4:**
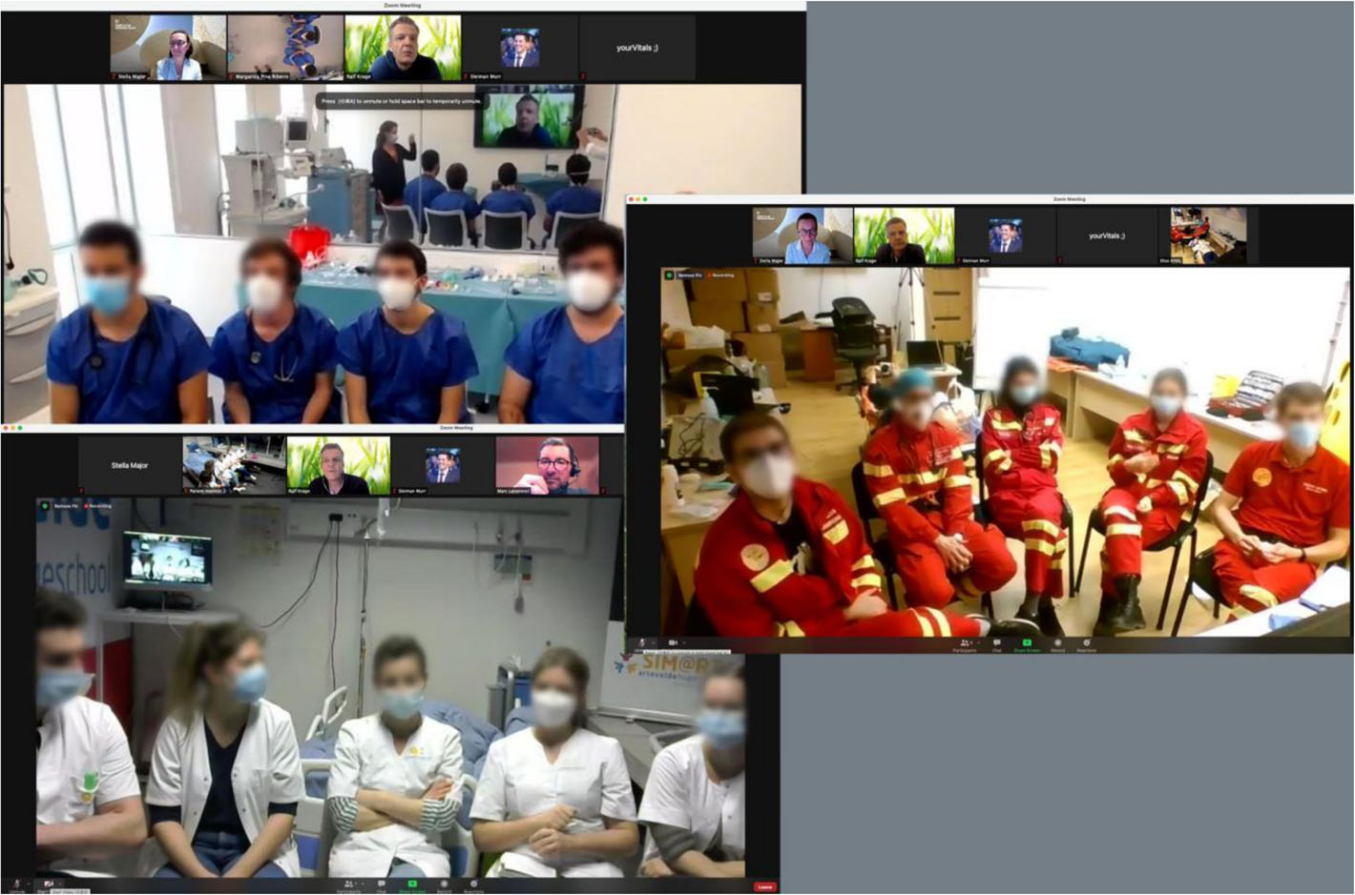
Debriefing sessions, as was visible to the facilitators

The end of the scenario was clearly announced by the debriefers after which the team was asked to sit in a row in front of the main camera and monitor, where the debriefing took place (Fig. 4).

#### Debriefing (approximately 15–20 min)

Debriefing was conducted immediately after the simulation session, in order to capture live reactions from the participants and minimize team self-debriefing. During debriefing, the whole team was visible to the debriefers, and the two debriefers were visible to the team on their respective screens. No video footage from the scenario could be used in the debriefing. We deemed it important to pay close attention to all reactions expressed by the students, given that the debriefers were at a remote location and might have missed nuances of non-verbal communication.

#### Wrap-up (approximately 5–7 min)

After thanking the participants, they were given further information on the next steps regarding the competition.

### Competition rating process

Throughout the competition (preliminary heats and finals), the teams were rated on their performance across two key domains:

1. Their technical and non-technical performance during the scenario
2. Their capacity to be self-reflective during the debriefing

Based on these criteria, once all the preliminary heats were completed, the debriefers identified the four finalist teams. The rating process of the final round also followed exactly the same process.

For the purpose of continuity, at least one SimUniversity faculty member was present in all the simulations and live debriefings, in order for us to secure the necessary comparability of all the team performances.

### Technical setup

**Fig. 5:**
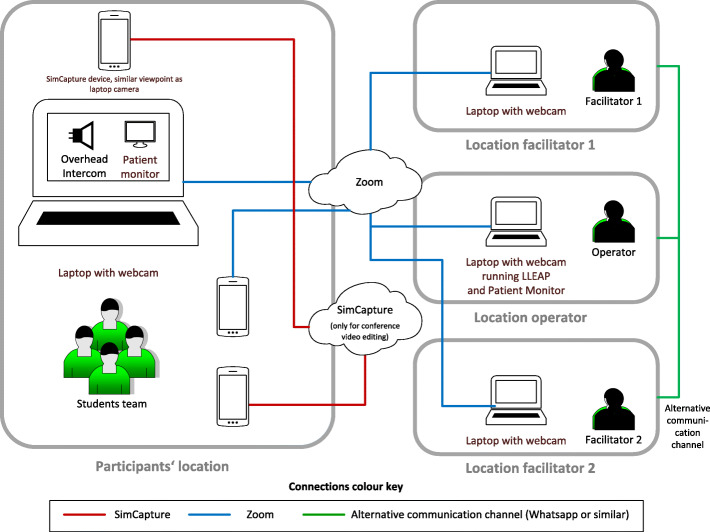
Schematic overview of the technological setup used

A complex technical setup was devised (Fig. 5) in order for the two separate goals to be achieved. Firstly, to support the facilitators’ remote simulation and debriefing and, secondly, to produce the video footage which would be shown within the virtual conference.

All participating student teams had to be in an environment which ensured a stable network connectivity, have access to appropriate clinical equipment, and also have a simulation manikin of any type. One set of mobile devices was connected to a Zoom call, offering the facilitators different viewing angles of the team and offering the team a view of the patient monitor (during the scenario case) and, later on, of the facilitators (during the debriefing). Additionally, throughout the entire event, all facilitators were also in communication with one another by a separate stable connection like WhatsApp, to communicate with one another, during the scenario and the debriefing. This was so that we would avoid using the built-in Zoom chat function, as it was deemed too risky, as we wanted to avoid unintentionally broadcasting the chat to everyone in the Zoom call.

In our setup, the second set of devices was used to record the session for later management in SimCapture and presentation at the virtual conference. The viewing angles of these devices were similar to the Zoom cameras. In addition, one handheld camera, held by a person not involved in the simulation case (present on site where the students were), was used to provide better insight and dynamics for the recorded scenario. It is important to mention that SimCapture was used for post-production video editing only in order to prepare the usage of the recorded material in the conference setting. If duplicating our setup to run a purely learner-centered synchronous event which does not need video recording or editing, Zoom would be sufficient. We opted for the recording because streaming such an event live could lead to a breakdown of technology, and this would be detrimental for conference attendees.

As emphasized by other authors who describe remote simulations, attention to sound quality was of utmost importance also in our setup [8]. In order to achieve this, a test session was scheduled with every team, during which time careful preparations were made to ensure good audio quality, absence of echoes, and mutual comprehensibility of team and facilitators

### Evaluation

To evaluate the effectiveness of the telesimulation, after each simulation session, the participants were sent an email, with a link to a voluntary and anonymous survey created in Qualtrics. They rated the effectiveness of the pre-brief, simulation, and debrief by responding to the simulation effectiveness survey (SET-M) items, using a 3-point Likert scale (strongly agree, somewhat agree, do not agree) in addition to providing optional free-text comments [10].

## Results

**Table:** 

	Preliminary simulations	Final simulations	Total number of surveys sent
**No. of students**	**38**	**19**	**57**
**SET-M survey responses**	**16**	**10**	**26 (46%)**

The SET-M survey was completed 26 times yielding a response rate of 46%.

### Pre-briefing

All respondents strongly agreed that the pre-briefing increased their confidence.

### Post-simulation

The majority of respondents (84%) strongly agreed that they had an opportunity to practice clinical decision-making skills. Similarly, 80% strongly agreed that they felt more empowered to make clinical decisions.

Three quarters (76%) strongly agreed that they felt more confident in their ability to report information to another member of the healthcare team, and 68% strongly agreed that the simulation made them feel better prepared to respond to changes in their patient’s condition and felt more confident to prioritize care and interventions.

One participant remarked: “This was one of the first times I felt like a physician, at the same time I felt already a better physician somehow.”

Another remarked on how the competition helped close gaps in their curriculum: “I learned a lot about why it is important to communicate and ‘close the loop’ [...] It’s something that hasn’t been regarded as important in our curriculum.”

### Debriefing

A majority (88%) of respondents strongly agreed that the debriefing allowed them to verbalize their feelings before focusing on the scenario, and 80% strongly agreed that debriefing provided an opportunity to self-reflect and that it was a constructive evaluation of the simulation. Furthermore, 76% strongly agreed that the debriefing contributed to their learning.

One student reflected on the challenge of competing in a language which they are not accustomed to. This is an important aspect for faculty to recognize and reinforce positively especially in the realms of remote simulations. As expected, we observed varying views about the technical issues that different participants experienced. One participant wrote: “I was a little skeptical about it being on zoom, but everything went fine.” Another wrote “Communication with the Voice of God wasn’t working all the time, but this could have been because of our technical limitations.” A third respondent was grateful despite the technical challenges and wrote: “I really enjoyed it! The remote control was a bit challenging but we couldn’t do (it) in other ways!”

## Discussion

Over the last decade, there has been a growing number of developments in remote simulation which have addressed various elements of the simulation as well as its technical setup. The pandemic further boosted this momentum, bringing about with its new terms and diverse technical structures and methodologies, thus addressing the gaps which resulted from either a halt in in-person education or to meet the need for rapid new skill acquisition of healthcare providers during the pandemic [3, 8, 10–12].

In order to prevent providers from misunderstandings, a clear terminology is needed when describing various aspects and setups of “distance learning.” According to the Healthcare Simulation Dictionary V2.0 Addendum, we used the term “remote simulation” for our setup [4]. As publications around this topic are rapidly rising, it seems that there is no unique language describing the same or at least very similar issues.

Duff et al. addressed this identifying terms like “distance simulation,” “telesimulation,” “remote simulation,” or even “virtual simulation” in the literature calling for a common blueprint for this new technique [8].

Remote simulation in general is considered feasible and effective [13]. It has been utilized to provide education, training, and assessment [11].

Treloar et al. examined the efficacy and feasibility of distance simulation-based learning in the military and found that using a manikin as a distance educational tool gives isolated medical personnel the opportunity to practice skills unconstrained by time or distance [14]. Reece et al. describe the benefits of using virtually facilitated simulation and virtual debriefing using the Zoom teleconference platform, for COVID-19 preparedness in Rural Canada. The virtually facilitated simulation was shown to be both a viable and cost-effective method of delivering simulation-based education during the COVID pandemic and effectively mobilized a team of interprofessional co-facilitators to support learners in geographically remote locations [15]. Other applications of remote simulation include both team-based training sessions in emergency medicine and neonatal resuscitation [16, 17] as well as procedural task training such as laparoscopic surgery and intraosseous needle insertion [12, 18].

Our approach to remote simulation was to provide both the student teams and the conference audience with a remote scenario and a virtually structured debriefing performed by experienced simulation educators as described by Ahmed et al. [19]. Virtual debriefings through a telecommunication platform potentially create unique challenges when comparing them with traditional in-person settings. Cheng et al. describe the three core elements that are necessary to create a “successful virtual learning environment.” These elements are *educator presence* (how educators design and implement educational activities, facilitate discourse, provide instruction), *cognitive presence* (the extent to which learners critically reflect and construct meaning through reflective discourse), and *social presence* (how learners project their personal characteristics) [3]. According to the authors, these three elements are closely linked and interrelated and offer a “valuable organizing principle for practical guidance.” In our setup, we addressed all three of these elements to offer the best possible experience for both the learners and the conference audience, under the given circumstances.

While we do not advocate for remote simulation to replace face-to-face experiences, the experience of the remotely facilitated session during the pandemic opens the horizon for us to include many more learners from more distant locations, into such educational conferences and events.

As we describe a novel approach to a subject currently undergoing a rapid growth, we summarize our lessons learned as follows:

1. A careful preparation, including a technology precheck with each team, is a key component of such an endeavor. Participants’ feedback also reflects the technical barriers that they faced. Given the geographic spread, the variability in employed technology, and other potential variables, the lack of preparation will directly impact the simulation session. Also, due to the remote character of the simulation, some problems cannot be quickly resolved, further underlining the necessity of a precheck.
2. Employing a separate simtechnologist (as depicted in Fig. 5) for controlling the vitals is highly recommended. In our setting, we did not employ a third facilitator as a simtechnologist in every session due to the conflicting schedules of the faculty. Thus, on several sessions, one of the facilitators (ML) played a dual role, also controlling the vitals, which led to a task overflow and sometimes caused delays in them providing timely replies to the team’s questions (overhead intercom).
3. Our own experience during the debriefing sessions as well as the results from the survey indicate that this type of remote simulation managed to achieve the set goals, i.e., to come as close as possible to a face-to-face simulation session which is followed by an immediate debriefing. Participants reported high levels of engagement, self-reflection, and learning both during the scenario and from the debriefing. While the relatively low response rate of 46% might mask a lower engagement or learning in the non-respondents, the general feeling of engagement both during scenario and debriefing, as perceived by the team of facilitators, would not support this hypothesis.
4. Ensuring psychological and physical safety is foundational to high-quality simulation-based training. Transferring this concept into the remote simulation is certainly a challenge, and the methods used depend to a certain degree on the exact type of distance simulation employed. In our case, as we tried to maintain as many aspects of classical simulation training as possible, we had to ensure different aspects of psychological safety. Firstly, all aspects around the usage of the video material were addressed, as would be the case in face-to-face training, prior to engaging the learners, and consent was obtained from each and every learner.
  Secondly, the safety of the physical environment of the learners was addressed through different methods. The facilitators always emphasized during the introduction that only minimal defibrillator energy should be used, and appropriate safety should be observed when working with sharp materials. This aspect remains, though a somewhat weak point of telesimulation in the way described in this paper, as the facilitators have no direct means of controlling the physical safety of the environment. In hindsight, this process might have been made more robust by asking the local coordinator, if available, to check for safety as would be the case in an in-person training activity. It is important to emphasize the relevance of physical safety to all participants and to keep the attention of the faculty on this aspect during the whole session.
  Thirdly, the safety and inclusiveness of the debriefing were respected as would be the case in a real face-to-face debriefing. Having all participants sitting in front of one camera allowed the debriefers the traditional view of the whole team, relatively similar to what they would see in a face-to-face debriefing. While also somewhat challenging, in our opinion, it is feasible for an experienced facilitator to lead a debriefing very much similar to a face-to-face setting. While we did not encounter any such situation, we would hypothesize that should a very emotional situation arise, where in the face-to-face setting, a personal conversation might be needed, this would be a great challenge in the virtual setting. We believe that this should be a relevant point for the faculty to discuss and agree upon in planning such an activity, such as a phone call or email, to the participants’ post-event, to ensure that the learners’ emotions are validated and further addressed.
5. While SimUniversity is designed and advertised as a truly interprofessional event, in reality, the majority of the teams consisted of only medical students. The facilitators decided to accept this and not exclude those teams based on our common experience of the difficulties of interprofessional simulation. Those difficulties are multifaceted but are based in the essence of a certain disconnection between medical and nursing schools [20]. In the takeaway messages at the end of debriefing, the interprofessional teams all gave a feedback describing the clear advantages of training together and learning to understand the roles of the other professions involved. This does only add to the motivation of the SimUniversity team to grow the percentage of interprofessional teams in future events.
6. There were some lessons to be learned from our interactions with the different teams as well. We feel that the requirement for gathering an interprofessional team could have been communicated more clearly and earlier, as this might have increased the number of truly interprofessional teams.
7. We also learned that defining one contact person, who then in turn keeps the rest of the team informed, is more helpful than communicating with all team members from afar. If available, the local coordinator of the team would be an ideal contact person, or alternatively, a member of the team could serve as the spokesperson. As the whole process is heavily based on asynchronous communication, streamlining communication processes is of paramount importance.
8. In addition to deriving participants’ immediate reactions through the SET-M survey, we also contacted some of the teams directly a few weeks after the competition. Our aim in doing so was to better understand the experiences of the participants, especially in order to adapt the structure for future similar events. This post hoc approach was met with great enthusiasm by the students, and we got valuable feedback and insights. Our takeaway from this was that a general follow-up contact should be performed with all teams, several weeks after the event, and this can be easiest when a team spokesperson or contact has been identified.

## Conclusion

Based on our experience and insights gained from this pilot project, we conclude that running a remote simulation competition is both feasible and achievable and can provide a meaningful opportunity for undergraduate medical and nursing students to increase their confidence in working as a team in caring for their patients. While the authors do not recommend this method to be superior to a face-to-face experience, we propose this model to be an alternative method to consider when educators are faced with imposed restrictions such as what we faced during the COVID-19 pandemic.

## Supplementary Information

**Additional file 1.**

## Data Availability

The quality assurance survey data is available from the corresponding author on reasonable request.

## Abbreviations

ABCDE: Airway-breathing-circulation-disability-exposure
SESAM: Society in Europe for Simulation Applied to Medicine
SET-M: Simulation Effectiveness Tool Modified
IFMSA: Federation of Medical Students Associations
EMSA: European Medical Students Association
ALS: Advanced life support
AMS: Altered mental status
iOS: iPhone operating system
LLEAP: Laerdal Learning Application
