# Supplementary material for: SimUniversity at a distance: a descriptive account of a team-based remote simulation competition for health professions students
Source: Adv Simul (Lond). 2022 Feb 8;7:6. doi: 10.1186/s41077-021-00199-5 (PMC8822656; doi:10.1186/s41077-021-00199-5)
Supplement: Supplementary file 1 — Additional file 1. [file 41077_2021_199_MOESM1_ESM.pdf]

Appendix 1

Technical and material overview

Medical equipment

- Stretcher or bed for simulator
- Airway equipment suitable for learners' level
- Defibrillator with monitoring capabilities
- I.v. equipment
- (fake) medication, one bottle of saline might do
- Syringes of different sizes
- Medication labels
- Stethoscope
- Bandages
- Immobilisation equipment (cervical collar, splints, etc)

Simulation equipment

- Manikin (any type, not even necessarily full-body)

IT technology

1. Simulation site
    a. Laptop with webcam (ideally external via USB), loudspeaker and (ideally) big external screen
    b. 1-2 other network devices for Zoom (mobile devices)
2. Facilitators
    a. Laptop with webcam, one of which can run LLEAP
